# Controllable tip exposure of ultramicroelectrodes coated by diamond-like carbon via direct microplasma jet for enhanced stability and fidelity in single-cell recording

**DOI:** 10.1038/s41378-024-00819-w

**Published:** 2025-01-23

**Authors:** Zhiyuan Du, Qingda Xu, Ye Xi, Mengfei Xu, Jiawei Cao, Longchun Wang, Xiuyan Li, Xiaolin Wang, Qingkun Liu, Zude Lin, Bin Yang, Jingquan Liu

**Affiliations:** 1 National Key Laboratory of Advanced Micro and Nano Manufacture Technology, Shanghai Jiao Tong University, Shanghai, China; 2 DCI Joint Team, Collaborative Innovation Center of IFSA, Department of Micro/Nano Electronics, Shanghai Jiao Tong University, Shanghai, China

**Keywords:** Electrical and electronic engineering, Bionanoelectronics, Biosensors

## Abstract

Precise and long-term electroanalysis at the single-cell level is crucial for the accurate diagnosis and monitoring of brain diseases. The reliable protection in areas outside the signal acquisition points at sharp ultramicroelectrode (UME) tips has a significant impact on the sensitivity, fidelity, and stability of intracellular neural signal recording. However, it is difficult for existing UMEs to achieve controllable exposure of the tip functional structure, which affects their ability to resist environmental interference and shield noise, resulting in unsatisfactory signal-to-noise ratio and signal fidelity of intracellular recordings. To address this issue, we chose a dense and electrochemically stable diamond-like carbon (DLC) film as the UME protection coating and developed a method to precisely control the exposed degree of the functional structure by directly fixed-point processing of the UME tip by the strong site-selectivity and good controllability of the atmospheric microplasma jet. By analyzing the interaction between the microplasma jet and the UME tip, as well as the changes in the removal length and microstructure of UME tips with processing time, the exposed tip length was precisely controlled down to the submicron scale. Biocompatibility experiments, electrochemical aging tests and real-time intracellular pH recording experiments have demonstrated that the DLC-UME with effective tip protection processed by microplasma jet has the potential to enable long-term detection of intracellular high-fidelity signals.

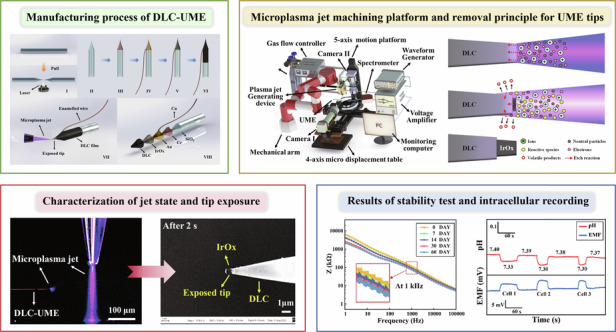

## Introduction

The development of implantable brain-computer interface devices is of increasing significance for the research of neurological diseases^1–3^. To obtain more accurate information from neurons, implantable-computer interfaces have been developed with devices with an ultra-micron scale facing the intracellular environment^4^. Reliable detection and analysis of intracellular chemical and biomarker signals at the single-cell level is crucial for early disease diagnosis, pathological assessment, and a deeper understanding of basic biology^5^.

Compared to microelectrodes, UMEs with a diameter of less than 10 μm have many advantages such as high spatiotemporal resolution, targeted precise regulation of neurons, and good biocompatibility^6^. Nanopipettes^7–9^, nanotubes^10,11^, nanowires^12,13^, and the sharp tip UMEs^14,15^ have received great attention in as minimally invasive intracellular electrophysiological detection^16,17^. Among those, sharp tip UMEs exhibit higher detection sensitivity for intracellular signals due to the modification of their tips by nano metal particles^18,19^. They can not only detect monomolecular-layer and sub-single molecule layer adsorbed on rough metal surfaces^20^, but also target specific biological or chemical signals of subcellular regions in single-cells for detection, such as pH (ref. ^21^), RNA (ref. ^22^), O_2_ (ref. ^23^), dopamine (DA) (ref. ^24^), reactive O species (ROS) and nicotinamide adenine dinucleotide (NADH) (ref. ^25^), etc.

The performance of implantable ultramicroelectrodes depends on their ability to transfer charges between patch clamp probes and cells. The reliable protection of UMEs has important effects on its signal-to-noise ratio and stability^14,26^. Commonly, researchers heat wax to adhere to the outer surface of the UMEs as a sealing layer owing to its characteristics of stable electric insulation capability, good airtightness and waterproof ability and short film-forming time and simple operability^27,28^. However, the thickness and coverage quality of the wax layer on UMEs are difficult to control^29^, which can lead to poor repeatability and stability of detection signals or the exposed length of the UME tips functional layers to be sometimes large and sometimes small. Excessive tip exposure will result in a portion of the sensing surface remaining outside of the cell^14^ and the UME being susceptible to environmental interference when recording intracellular signals, increasing unknown noise signals and weakening its ability to detect targeted sites at specific location of the cell, affecting signal fidelity, while the naturally formed small exposed length of the tip functional layer after wax encapsulation^30^ or the circular electrode structure formed by precision polishing methods^15,31^ will lead to a small contact area between the tip functional layer and the intracellular environment during the implantation. Due to the inverse ratio between the impedance and surface area, the impedance of the UME is too high which will affect its charge transfer ability, reducing the signal-to-noise ratio and weakening its ability to distinguish low amplitude signals^32^. Some researchers manufactured carbon nanocone electrodes by pyrolyzing carbon into nanopipettes, and then used HF to etch the quartz protective layer outside the electrode tip, exposing the carbon nanotips^33,34^. Some researchers inserted SiC nanowires modified with nanoparticles^25,35–37^ or molecular crystal waveguides^38^ into glass micropipettes filled with liquid metal and wax to control the exposed length and achieve effective insulation. Unfortunately, the above methods still suffer from issues with excessive or uncontrollable exposed tip length.

The cold atmospheric microplasma jet has been applied in the fields of biology and medicine in recent years^39,40^ due to its characteristics of low temperature, no mask required, strong site selectivity, simple operation, high efficiency, and low cost^41,42^. It has enormous potential for micro and nano scale surface microfabrication. However, to the best of our knowledge, there is still limited literature on the application of the microplasma jet in the treatment of ultra microelectrode tips, in which the microelectrodes were only subjected to surface roughening treatment^43^ or a low precision and uncontrollable processing^44^. None of them can be applied to single-cell analysis.

Here, we describe a method of selectively etching UME tip protective coatings using microplasma jet, enabling controllable exposure of tip functional coatings to achieve effective insulation and interference shielding. Due to the favorable characteristics such as superior mechanical properties, high thermal stability, biocompatibility, biochemical inertness, resistance to biofouling and biocompatibility of the DLC^45,46^, we choose DLC film as a protective coating for the sharp UME for the first time. We test the emission spectra of different reaction gases and compare the site-selectivity of microplasma jets on UMEs under different processing modes, characterize the UME tips before and after depositing DLC coatings by scanning electron microscope (SEM). By analyzing the variation of the removal length of the DLC coating on the UME tip with processing time and the corresponding SEM micrographs, the exposed length of the UME tip is well controlled, down to submicron scale. Then, we evaluate the phase composition and lattice orientation of the DLC film deposited on the UMEs, analyze the elemental composition and compositional changes after microplasma jet processing and verify through a one-week biocompatibility control experiment that the DLC-UME has no adverse effects on the normal growth of neuron cells. Finally, electrochemical aging tests and intracellular pH detection experiments provide evidence that the DLC-UME has good electrochemical stability and can detect high-fidelity signals in the single cell in real-time. Our work provides a new method for the external effective protection of UMEs in single-cell analysis, which can expand more available materials as the protective layers of UMEs and achieve controllable exposure of UME tips to enable the precise and long-term detection of intracellular substances.

## Materials and methods

### Platform for microplasma jet machining

The platform for microplasma jet processing of UMEs comprises a microplasma jet generator made by a 3D printer (MicroArch S240A, BMF Precision Tech Inc., China), a high voltage amplifier (Trek Model 30/20 A, Trek, Inc., USA), a waveform Generator (SDG2042X, SIGLENT, China), a mechanical arm with six degrees of freedom (JAKA Zu 3, JAKA Robotics Co., Ltd., China), an observation camera (MER2-2000-19U3C, DAHENG IMAGING, China), a visual positioning camera (JAKA Lens 2D, JAKA Robotics Co., Ltd., China), a five-axis motion platform with three moving axes and two rotating axes (Shanghai Cheng Fang optical instrument co., LTD, China), a spectrometer with a fiber optic probe (QEPRO-VIS-NIR, Ocean Optics (SHANGHAI) Co., Ltd., China), a gas flow controller, two high pressure gas cylinders, and a central-control personal computer. A quartz capillary (Outer diameter: 1 mm and inner diameter: 0.2 mm, Sutter Instrument Co., Novato, CA, USA) was pulled into a nozzle with an inner diameter of 8 μm by the CO_2_ laser- puller (P2000, Sutter Instrument Co., Novato, CA, USA) to generate the microplasma jet. A copper ring with a length of 6 μm was fixed around the nozzle as the high voltage electrode with 8 cm to the end of the nozzle. The UME was fixed on a silicon wafer deposited with a DLC film of the same thickness as it.

### Characterization method of the DLC-UME

SEM micrographs and energy dispersive spectroscopy (EDS) of the UME tip processed by microplasma jet were collected by the super resolution field emission scanning electron microscope (JSM-7800F, JEOL Ltd., Japan). Raman spectrum of UME tips before and after microplasma jet processing was collected by the Raman images-SEM combined instrument (RISE-MAGNA, TESCAN CHINA, Ltd., Czech Republic). X-ray diffraction (XRD) spectra of the DLC coating on the surface of the processed UME was collected by multifunctional X-ray diffraction (D8 ADVANCE Da Vinci, Nasdaq: BRKR, Germany).

### Biocompatibility testing method

The resuscitated HT22 cells were cultured in DMEM containing 1% penicillin (100 IU/mL) - streptomycin (0.1 mg/mL) and 10% FBS in a 5% CO_2_ incubator at 37 °C. To verify the biocompatibility of the UME coatings, a UME was placed it into a culture medium containing HT22 cells transfected with fluorescence of GFP, and the growth status of cells under bright background, fluorescent background and mixed background were observed through controlled experiments by an inverted microscope (IX73, Olympus Corporation, Japan).

### Reagents for electrochemical testing

The fetal bovine serum (FBS), antibiotics, hippocampal neuronal cell line (HT22), and Dulbecco’s Modified Eagle Medium (DMEM) were purchased from Sigma-Aldrich (Shanghai, China). Trypsin-EDTA (0.25%) was purchased from Thermo Fisher Scientific Inc. (Waltham, US). Cell freezing medium was purchased from Biosharp (Hefei, China).

### Electrochemical testing method

Cyclic voltammetry (CV) tests in the range of −0.6 V to 0.8 V with scanning rate of 100 mV/s, and electrochemical impedance spectroscopy (EIS) tests in the range of 1 Hz to 100 kHz of the DLC-UME were performed in 10 mM phosphate buffer saline (PBS) at pH 7.4 by a standard three-electrode system (PGSTAT204, Autolab, Switzerland).

### Intracellular data collection and signal processing method

The electric micromanipulator (TransferMan 4r, Eppendorf, Germany) was used to control the UME to approach and implant into single cells. The intracellular and extracellular EMF curves in DMEM were collected by the analog-to-digital converter (Molecular Devices Digidata 1440 A, USA) and the amplifier (MultiClamp 700B, Molecular Devices, USA), processed by the software (Molecular Devices, pCLAMP 10), and output by the software (Clampfit 10.7).

## Results and discussion

### Fabrication method of the DLC-UME

**Fig. 1:**
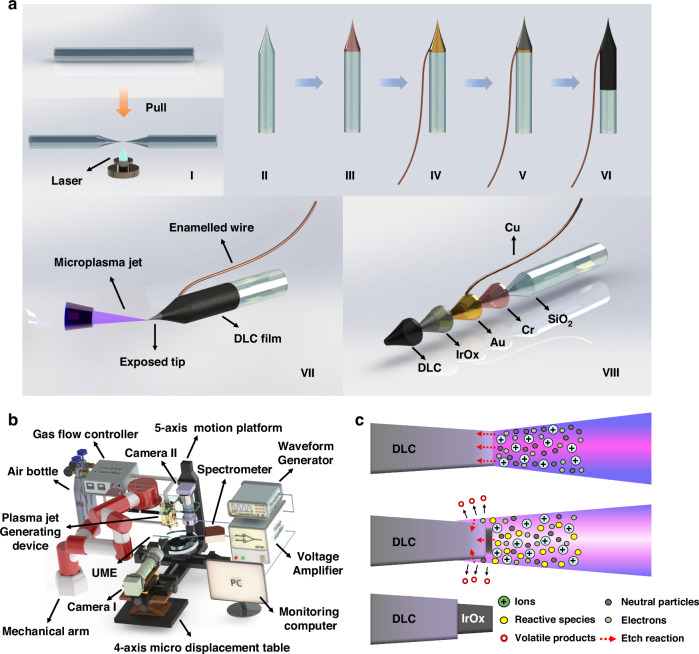
Fabrication of the DLC-UME and principle of microplasma jet processing for UME tips. **a** Fabrication of the DLC-UME: **a-I** Laser-assisted pulling of quartz capillary into nanopipette. **a-II** The nanopipette after pulling. **a-III** Sputter Cr layer. **a-IV** Sputter Au layer and leading enameled copper wire. **a-V** Sputter IrOx layer. **a-VI** Depositing DLC layer. **a-VII** Exposing the UME tip by microplasma jet. **a-VIII** Distribution of DLC-UME coatings. **b** Platform for microplasma jet processing of UMEs. **c** Principle of removing DLC coating at UME tip by microplasma jet

A quartz capillary (Outer diameter: 1.0 mm and inner diameter: 0.7 mm, Sutter Instrument Co., Novato, CA, USA) was pulled into two nanopipettes assisted by CO_2_ laser-puller (P2000, Sutter Instrument Co., Novato, CA, USA) with appropriate pull parameters (Heat: 950, Velocity: 50, Delay: 168, Filament: 2, Pull: 120) (Fig. 1a-I). Cr and Au were sputtered onto the nanopipette surface at the power of 105 W and the chamber pressure of 1 Pa for both 1 min. The thickness of the Cr layer was about 10 nm and the thickness of the Au layer was about 40 nm. An enameled Cu wire was fixed on the Au layer with conductive silver paste and further baked in a vacuum drying oven at 120 °C for 30 mins until the paste solidifies (Fig. 1a-II, III, IV). An Ir layer was sputtered onto the Au layer as the seed layer of iridium oxide (IrOx) at the power of 105 W with an Ar flux of 13 sccm and an IrOx layer was further sputtered onto the Ir layer at the power of 105 W with an Ar flux of 13 sccm and an O_2_ flux of 13 sccm (Fig. 1a-V). The thickness of the IrOx layer was about 200 nm. Then, a DLC layer with a thickness of about 400 nm was deposited onto the IrOx layer by pure ion coating technology (Anhui Chunyuan Coating Technology Co., Ltd, China) (Fig. 1a-VI) and removed by microplasma jet to expose the IrOx layer on the UME tip (Fig. 1a-VII). The surface coating distribution of the processed UME is shown in Fig. 1a-VIII.

### Processing of microplasma jet treatment for UMEs

Under the monitoring of the positioning camera and the observation camera in real-time, the angle of the nozzle was controlled through the robotic arm, and the position of the UME tip was adjusted through the five-axis motion platform to be about 100 μm below the nozzle. Then, A 2 kHz sinusoidal voltage output by the waveform generator was amplified to peak-to-peak voltage of 12 kV to 14 kV by the high voltage amplifier and applied to the ring electrode (Fig. 1b).

### Principle for the microplasma jet removal of the DLC coating on the UME tip

The DLC coating contains diamond structure and graphite structure, with carbon atoms mainly bound by sp_3_ and sp_2_ hybrid bonds. Therefore, it will be damaged by the physical interaction of microscopic particles in the plasma and will also undergo chemical reactions with reactive species such as O plasma, producing volatile products (Fig. 1c). Electrons and ions (such as He) in microplasma jet can be accelerated by the electric field to be directly bombarded on the DLC surface, leading to the breakdown of covalent bonds on the DLC-UME surface and activation of the DLC. Then, the activated surface easily reacts with O free radicals to introduce O-containing functional groups. After microplasma treatment^43^. These two processes together lead to the removal of the DLC coating and the changes in the surface morphology of the UME tip.

### Selection of the reaction gas and the horizontal machining distance

**Fig. 2:**
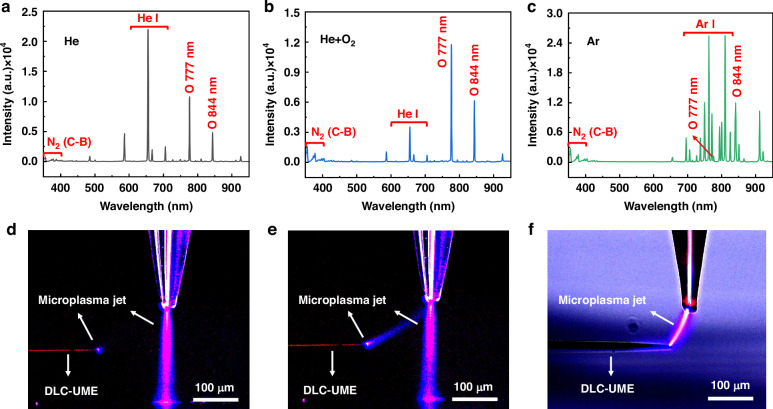
Optical emission spectrums of the microplasma jet acting on UME tips under different reaction gases and interaction states between the microplasma jet and the DLC-UME under different horizontal spacing. **a** Optical emission spectrum of He microplasma jet under the He flow rate of 20 sccm. **b** Optical emission spectrum of He/O_2_ microplasma jet under the He flow rate of 20 sccm and the O_2_ flow rate of 2 sccm. **c** Optical emission spectrum of Ar microplasma jet under the Ar flow rate of 20 sccm. **d** Interaction state under maximum horizontal spacing. **e** Interaction state under medium horizontal spacing. **f** Interaction state under smaller horizontal spacing

The species generated in the microplasma jets acting on UME tips under different reaction gases was detected by the optical emission spectrum using the spectrometer with a fiber optic probe (Fig. 2a–c). The spectrum of He microplasma jet under the He flow rate of 20 sccm was dominated by neutral helium atoms lying in the wavelength range of 550–750 nm. Reactive O atoms were also found at 777.7 nm, which indicated that the air in the jet path also undergoes a certain degree of ionization to generate reactive O species during the process of He microplasma jet generation (Fig. 2a). After mixing a flow rate of 2 sccm of O into helium, significantly reactive O atoms at 777.7 nm dominated the spectrum and reduced the production of neutral helium atoms (Fig. 2b). These reactive O atoms mainly come from the collisions between O molecule and He* or the inelastic electron-impact collisions with O molecule^43^, and play a significant role in the removal and surface modification of DLC. As a comparison to the He microplasma jet, the spectrum of Ar microplasma jet under the Ar flow rate of 20 sccm was dominated by neutral argon atoms lying in the wavelength range of 700–850 nm with higher emission intensity and less reactive O atoms (at 777.7 nm) (Fig. 2c).

Due to the smaller atomic mass and lower glow ignition voltage of He compared to Ar, the reaction between the He microplasma jet and the UME is less intense and easier to control. Moreover, some O-containing functional groups from the O microplasma jet undergo thermal decomposition and react with DLC to generate small molecule gases, such as CO, CO_2_, and water vapor during activation processes^47^, thereby reducing the residue on the surface of UME. Therefore, in this work, we chose a mixed microplasma of He and O_2_. Due to O_2_ being a negatively charged gas, excess O_2_ can absorb electrons from microplasma jets, leading to a decrease in the amount of reactive O atoms and ultimately reducing the DLC removal rate and effect. So, the O_2_ flow rate was chosen to be 2 sccm here.

A He/O_2_ microplasma jet formed on the silicon substrate deposited with the DLC coating after dielectric barrier discharge. As the UME gradually approached the jet, a weak branch of the microplasma jet began to be attracted to the UME tip when the horizontal spacing was around 150 μm (Fig. 2d). This is because the motion of conductive particles causes the microplasma jet to focus on the position with the closest distance and the highest relative conductivity. At this moment, the jet intensity was not sufficient to remove the dense DLC coating on the UME surface. When the horizontal distance was around 80 to 120 μm, the jet intensity was just enough to slowly remove the DLC coating on the UME surface, resulting in better machining accuracy and controllability of the exposed tip length, which helps to process the UMEs with finer tips and the coatings with a wider range of available materials (Fig. 2e). When the horizontal distance was less than 50 μm, the entire jet with a greater intensity was directly focused on the UME tip, resulting in higher processing rate and lower processing accuracy, which is more suitable for processing the UMEs with the larger tip size and the protective coatings of larger thickness and harder-to-machining materials (Fig. 2f). Therefore, in this work, we placed the UME at a horizontal distance of 100 μm and used the branch of microplasma jet to process the UME tip.

### Characterization of the DLC-UME surface coatings

**Fig. 3:**
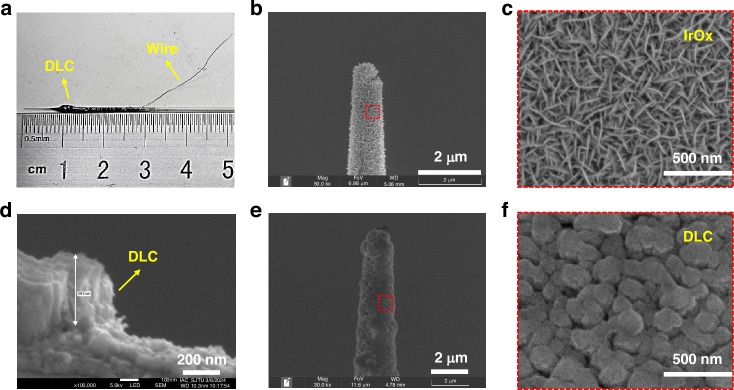
Photograph of the fabricated DLC-UME and SEM images of the surface coatings on the UME tips. **a** Photograph of the fabricated DLC-UME. **b** SEM image of the UME tip before deposition of DLC coating. **c** Magnified SEM image of IrOx coating on the surface of the UME tip in the red box in b. **d** SEM image of cross-section of surface coatings on the UME tip. **e** SEM image of the UME tip after deposition of DLC coating. **f** Magnified SEM image of DLC coating on the surface of the UME tip in the red box in e

The fabricated DLC-UME is presented in Fig. 3a. IrOx was chosen as the conductive layer for the UME because it is a typical material with Faraday pseudocapacitive properties and is compatible with MEMS processes, with good detection repeatability, electrochemical stability, biocompatibility, and targeted detection ability for intracellular pH^15,48^. The IrOx layer on the UME tip presented an earthworm-like nanowire structure, increasing the surface area of the UME detection point and reducing impedance (Fig. 3b and c). Due to the simultaneous reversible reactions between Ir^3+^ and Ir^4+^ states^49^, the capacitance of the UME increases and the ability of UME to transfer charges within cells is enhanced, thereby increasing detection sensitivity and signal-to-noise ratio. From the cross-section of the UME tip, the thickness of the DLC layer covering the IrOx was consistent with the expected thickness of about 400 nm (Fig. 3d). The DLC layer had a dense nanoparticle structure (Fig. 3e and f), which enabled good coverage of the the UME surface and contributes to the effective protection of the UME.

### Selection of processing time and control of processing effect for UME tip

**Fig. 4:**
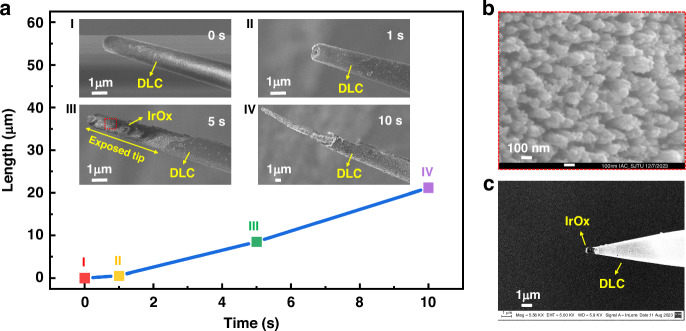
The relationship between the length of the etched tips and time, and the morphological changes of the UME tip during the etching process. **a** Relationship between the length of the etched tips and processing time: SEM images of the UME tips etched by microplasma with the processing time of **a-I** 0 s (untreated), **a-II** 1 s, **a-III** 5 s, **a-IV** 10 s. **b** Magnified SEM image of the UME surface in the red box in a. **c** SEM image of the processed UME tip with submicron exposed length. Microplasma jet parameters: He flow rate: 20 sccm, O_2_ flow rate: 2 sccm, peak to peak applied voltage: 13 kV

In this experiment, the controllable fixed-point processing of UME tips was achieved by controlling the processing time of the He/O_2_ microplasma jet branch with a diameter of approximately 3 μm. The processing time was kept at 1 s, 5 s, and 10 s, with the material removal length at the UME tip being 0.5 μm, 8.5 μm and 21.2 μm, respectively (Fig. 4a). Here, the relationship between removal length and exposed length was determined by the shape of the UME tip from the SEM pictures. We can see from Fig. 4a-I that the untreated UME tip was approximately semi-circular in shape. Compared with Fig. 4a-II, it can be seen that the UME tip was just exposed, so we infer that the removal length at this time was about half the diameter of the UME tip in Fig. 4a-I, which was 0.5 μm. Similarly, by measuring the exposed length of Fig. 4a-III and Fig. 4a-IV, and then adding 0.5 μm to this length, the removal length of other processing time was obtained. The approximate etching rate can be estimated by measuring the relationship between the removal length and the processing time, which was approximately 2.12 μm/s. The material removal length at the UME tip increased with increasing processing time. The DLC layer at the UME tip began to be removed after 1 s, exposing the inside IrOx layer, and the surface of the UME was slightly roughened (Fig. 4a-I, II). After a processing time of 5 s, the exposed tip length of the IrOx layer has exceeded 5 μm (Fig. 4a-III). At this time, the IrOx coating has also been subjected to surface modification and roughness to a certain extent by microplasma jet (Fig. 4b), and the surface area continued to increase, which helps to reduce the impedance of the UME and improve detection sensitivity. Furthermore, the excessive processing time of more than 10 s caused significant damage to the conductive layer material and the exposed length to be too large of the UME tip (Fig. 4a-IV). Therefore, the processing time should be controlled within 5 s. Here, we controlled the processing time of the microplasma jet to 2 s, and the exposed tip length of the processed UME was about 900 nm (Fig. 4c), achieving controllable processing of the UME tip with submicron resolution, which is ahead of the work of other researchers (Table S1).

### Composition, structure, and biocompatibility testing of processed DLC-UME

**Fig. 5:**
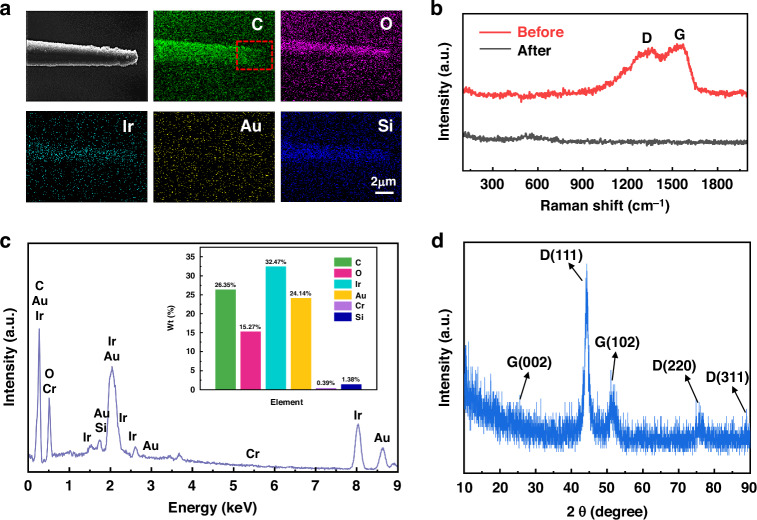
Testing of EDS, Raman spectrum, and XRD spectrum of processed DLC-UME. **a** Images of element distribution on the surface of the processed UME tip. **b** Raman spectrum of UME tips before and after microplasma jet processing. **c** EDS and element weight percentage of the processed UME tip. **d** XRD of the DLC coating on the surface of the processed UME

EDS showed that the DLC-UME was composed of C, O, Ir, Au, Cr and Si elements, with the weight percentage of 26.35, 15.27, 32.47, 24.14, 0.39, 1.38, respectively (Fig. 5c). Moreover, the atomic ratio of Ir to O is 4.83: 27.30. The relative content of each element is influenced by the thickness and distribution of each coating from the outside to the inside of the UME. By comparing the distribution and density of C atoms in the red box area of Fig. 5a with other elements, it can be concluded that the DLC coating at the UME tip has been successfully removed, and other coatings were still intact (Fig. 5a). The surface of the untreated UME tip was coated with DLC film with typical mixed structure of sp^2^ and sp^3^ carbon^50^, and its Raman spectrum consisted of the typical D-peak of 1350 cm^−1^ and G-peak of 1580 cm^−1^, while the presence of D-peak or G-peak was not detected at the UME tip after microplasma jet processing due to the complete removal of the DLC coating on the UME surface (Fig. 5b).

Then, five diffraction peaks were found at 2θ values of 26.2°, 44.1°, 51.6°, 75.4°and 89.1° in the XRD spectra of the processed UME (Fig. 4d). It can be observed that the XRD spectra was dominated by three intense peaks located at 2θ ∼ 44.1°, 75.4° and 89.1°, which can be identified by the reflection of diamond (111), (220) and (311) planes^50^. The peaks located at 2θ ∼ 26.2° and 51.6° were corresponding to the graphite (002) and (102), respectively^51^. In a word, the DLC coating on the surface of the processed UME was composed of a certain proportion of diamond graphite with high crystallinity. Furthermore, the growth status of the cells with the UME placed within seven days was as good as that of the control group within the seven days of testing, which indicated the DLC-UME has good biocompatibility (Fig. S1).

### Electrochemical characterization of DLC-UME

**Fig. 6:**
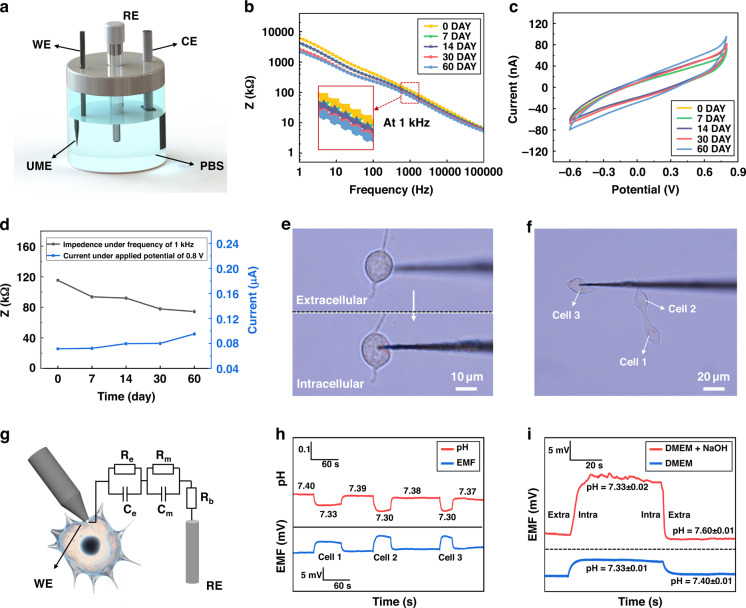
Tests of long-term electrochemical stability and experiments of real-time single-cell intracellular pH detection of DLC-UME. **a** Schematic diagram of three-electrode system for electrochemical characterization of the UME. **b** Impedance-frequency curves of the DLC-UME under different aging time. **c** CV curves of the DLC-UME under different aging time. **d** Curves of the changes in current and impedance within 60 days. **e** Microscopic images before and after UME insertion into a cell. **f** Microscopic images of three cells inserted by the UME. **g** Equivalent circuit diagram of the UME inserted into the cytoplasm in a dual electrode system. **h** Potential curves and corresponding pH curves of the DLC-UME when the UME is sequentially inserted from extracellular to intracellular of the three cells in DMEM in f. **i** Potential curves and corresponding pH value inside and outside the cell 1 before and after adding NaOH into the DMEM

The electrochemical performance was tested using a micromanipulator to clamp the DLC-UME as the working electrode (WE), an Ag/AgCl electrode as the reference electrode (RE), and a Pt electrode as the counter electrode (CE) (Fig. 6a). Accelerated aging testing has been used to evaluate the intracellular lifespan of UMEs^52^. Placing the UME in a high-temperature environment will accelerate the chemical reaction of the UME surface coatings to accelerate its degradation, thus evaluating the long-term stability of the UME in a short period of time^53^. A commonly used formula is Eq. S1^47^. In our work, the reference temperature was set to 37 °C suitable for HT22 cells growth and the ambient temperature was kept at 60 °C for an accelerated aging factor of 4.9 according to Eq. S1 by placing the beaker with electrodes on a hot plate. The impedance frequency response curves and CV curves of the DLC-UME were recorded with the accelerated aging time of 0 h, 34 h, 68 h, 147 h, and 294 h, corresponding to approximately 0 days, 7 days, 14 days, 30 days, and 60 days, respectively (Fig. 6b, c). As the UME ages, the impedance slowly decreased at the frequency of 1 kHz, and the limit current slowly increased at the applied potential of 0.8 V. The changes in impedance and current within 60 days were very small (Fig. 6d).

The electrochemical performance of the UME was still stable after 60 days, which may be attributed to the good electrochemical stability of the DLC coating and the modification of the DLC coating by microplasma jet. With the introduction of O-containing functional groups in the microplasma jet, sp_2_ bonding was broken, reducing the degree of graphitization of DLC^54,55^. O atoms adsorbed at defect sites in DLC material to form various O-containing functional groups and more sp_3_ carbon appears, resulting in a decrease in conductivity and an increase in internal Ohmic resistance of the DLC coating^56^, which may improve the compactness and the electrochemical shielding ability of the DLC coating and contribute to the effective protection of the UME.

### Electrophysiological recordings of intracellular signals of neurons

Electromotive force (EMF), which represents the potential difference between the RE and the WE at zero current, which can be used to characterize the intracellular potential response^57^. The DLC-UME was inserted into the cultured HT22 cells, and the cell membrane underwent slight deformation (Fig. 6e). Then, the UME was sequentially implanted into three different cells for the signal recording (Fig. 6f). Equivalent circuit diagram of the UME inserted into the cytoplasm in a dual electrode system is shown in Fig. 6g, where R_e_ represents electrode resistor, R_m_ represents cell membrane resistor, R_b_ represents bulk solution resistor, C_e_ represents electrode capacitor, and C_m_ represents cell membrane capacitor, which is used to simplify complex behaviors inside and outside cells^15^.

Intracellular pH value is closely related to cellular metabolism, carcinogenesis, and apoptosis, which is crucial for a deeper understanding and diagnosing diseases. The corresponding relationship between pH and EMF, named the E-pH response sensitivity, was measured to be approximately 60.92 mV per pH by placing the UME in solutions of different pH values (Fig. S2), which was closed to the Nernst response (57.80 mV per pH)^48^. The EMF curves and the corresponding pH curves of the DLC-UME were tested when the UME was sequentially inserted from extracellular to intracellular of the three cells in Fig. 6f in the same culture medium at 7.4 pH (Fig. 6h), which indicates that the DLC-UME has the ability to continuously and stably detect pH within different cells, which can be used to diagnose the health status of different cells.

The EMF difference between intracellular and extracellular cytoplasm in the cell 1 in Fig. 6f was approximately 4.26 mV. When a small amount of NaOH solution was dropped into the culture medium to increase the pH to 7.6, the potential difference increased to about 13.4 mV and the intracellular pH value within 60 s was still about 7.33, basically consistent with the value before adding NaOH solution (Fig. 6i). Due to the addition of a small amount of NaOH in the culture medium, the pH value of the surrounding environment of the cell increases, exceeding the optimal pH range for cell growth, which affected the acid-base balance inside and outside the cell. At this time, physiological activities of balancing osmotic pressure occurred inside the cell, and the pH and the EMF inside the cell underwent slight changes. Because the cell membrane was not damaged in a short period of time, the difference of the EMF inside and outside the cell remained stable. The implantation of the DLC-UME caused minimal damage and the cell membrane was not damaged, so the extracellular fluid did not penetrate into the cell, and the cell was not affected by changes in the pH of the culture medium in a short period of time. Results above demonstrated that the DLC-UME was sensitive to pH changes and had good reversibility and stability in intracellular recording.

## Conclusion

This work presented a method of selectively etching UME tip protective coatings using microplasma jet, enabling controllable exposure of tip functional coatings to achieve effective insulation and interference shielding. DLC coating was chose as a protective layer for the sharp UME for the first time. By determining the optimal interaction mode between microplasma jet and UME and analyzing the changes in the microscopic morphology of UME tips with processing time during the removal of protective layer materials, the exposed tip length was precisely controlled down to the submicron scale. Then, we evaluated the phase composition and lattice orientation of the DLC film deposited on the UMEs, analyzed the elemental composition and compositional changes after microplasma jet processing and verified through a one-week biocompatibility control experiment that the DLC-UME had no adverse effects on the normal growth of neuron cells. Finally, electrochemical aging tests and real-time single-cell intracellular pH detection experiments provided evidence that the DLC-UME with effective tip protection processed by microplasma jet held the potential to enable the precise and long-term detection of intracellular signals. Our work provides a new method for the external effective protection of UMEs in single-cell analysis, which can expand more available materials as the protective layers of UMEs, improve the fidelity and long-term stability of single-cell recording and contribute to the scientific research on accurate diagnosis and monitoring of brain diseases in the future.

## Supplementary information

Supporting file

## Data Availability

The authors declare that all data supporting the findings of this study are available within the paper and its Supplementary information. The raw data acquired in this study are available from the corresponding author on reasonable request. The main data supporting this study’s results are available within the paper and its supplementary information. Source data are provided with this paper. Relevant information is available from the corresponding author.
