# Supplementary material for: Controllable tip exposure of ultramicroelectrodes coated by diamond-like carbon via direct microplasma jet for enhanced stability and fidelity in single-cell recording
Source: Microsyst Nanoeng. 2025 Jan 23;11:20. doi: 10.1038/s41378-024-00819-w (PMC11754621; doi:10.1038/s41378-024-00819-w)
Supplement: Supplementary file 1 — Supporting file [file 41378_2024_819_MOESM1_ESM.docx]

Supplementary Information for

Controllable tip exposure of ultramicroelectrodes coated by diamond-like carbon via direct microplasma jet for enhanced stability and fidelity in single-cell recording

Zhiyuan Du^1,2^, Qingda Xu^1,2^, Ye Xi^1,2^, Mengfei Xu^1,2^, Jiawei Cao^1,2^, Longchun Wang^1^, Xiuyan Li^1^, Xiaolin Wang^1^, Qingkun Liu^1^, Zude Lin^1^, Bin Yang^1^, and Jingquan Liu^1*^

^1^ National Key Laboratory of Advanced Micro and Nano Manufacture Technology, Shanghai Jiao Tong University, Shanghai, 200240, China and

^2^ DCI Joint Team, Collaborative Innovation Center of IFSA, Department of Micro/Nano Electronics, Shanghai Jiao Tong University, Shanghai, 200240, China

*Corresponding author: Jingquan Liu

Email: [jqliu@sjtu.edu.cn](mailto:jqliu@sjtu.edu.cn); Tel: 86-21-34207209; Fax: 86-21-34206883

**Table S1**

**Table S1. Comparison with previous works in recent researchers.**

| **Author** | **Exposed tip size** | **Controllability** | **Insulation** | **Encapsulation method** |
| --- | --- | --- | --- | --- |
| Pan et al. [28] | Random | - | + | Inserted into **wax** solution |
| Hunt et al. [29] | **20 μm** in length |  |  |  |
| Roberts et al. [14] | **5 μm** in length | + | - | Placed into **phenolic** solution for electro-polymerization |
| Gao et al. [31] | Only a **ring** on the cross-section | - | ++ | Inserted into **wax** solution and mechanical polish |
| Xu et al. [15] | Only a **ring** on the cross-section | - | ++ | Inserted into **wax** solution and grind by diamond disk |
| Zhang et al. [35] | About **5 μm** in length | + | ++ | Inserted into **glass** micropipette and **wax** solution |
| Wu et al. [36] | **4 μm** in length |  |  |  |
| Jiao et al. [25] | **5 μm** in length |  |  |  |
| Jiang et al. [37] | **10 μm** in length |  |  |  |
| Xu et al. [38] | **28 μm** in length | + | - | Inserted into **glass** micropipette micromanipulator |
| Xi et al. [44] | **33 μm** in length | - | - | Deposited with **parylene-C** and etched by plasma jet |
| This work | **0.8 μm** in length | ++ | ++ | Deposited with **DLC** and etched by microplasma jet branch |

Technique performance in each work is ranked as follows: –, poor; +, fair; ++, good.

**Figure S1**

**
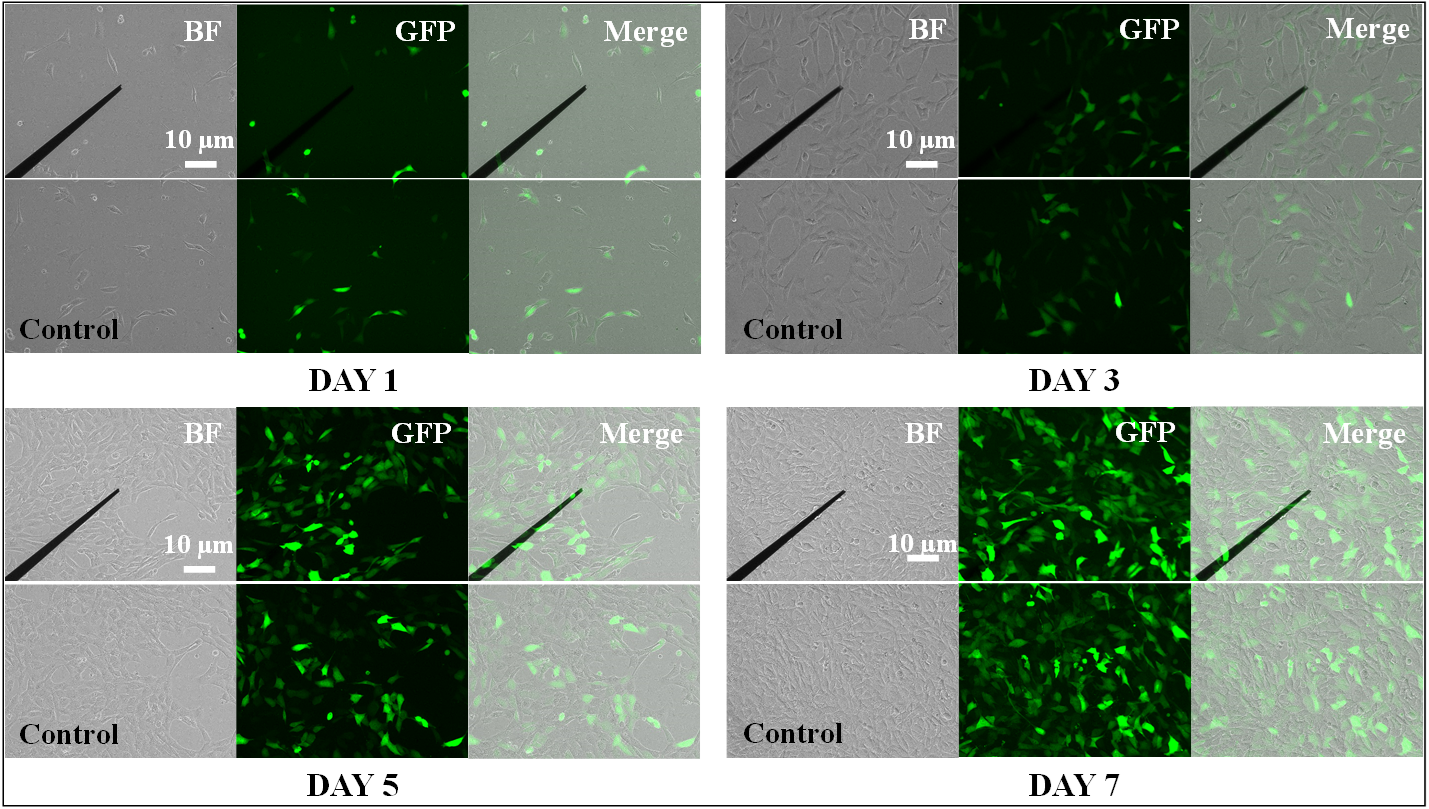
**

**Fig. S1: Biocompatibility testing of the DLC-UME within 7 days.**

**Equation S1**

**Eq. S1: Formula for accelerated aging of UMEs.**

*f* =2^ΔT/10^

Δ T is equal to the difference between the ambient temperature during electrochemical testing and the reference temperature.

**Figure S2**


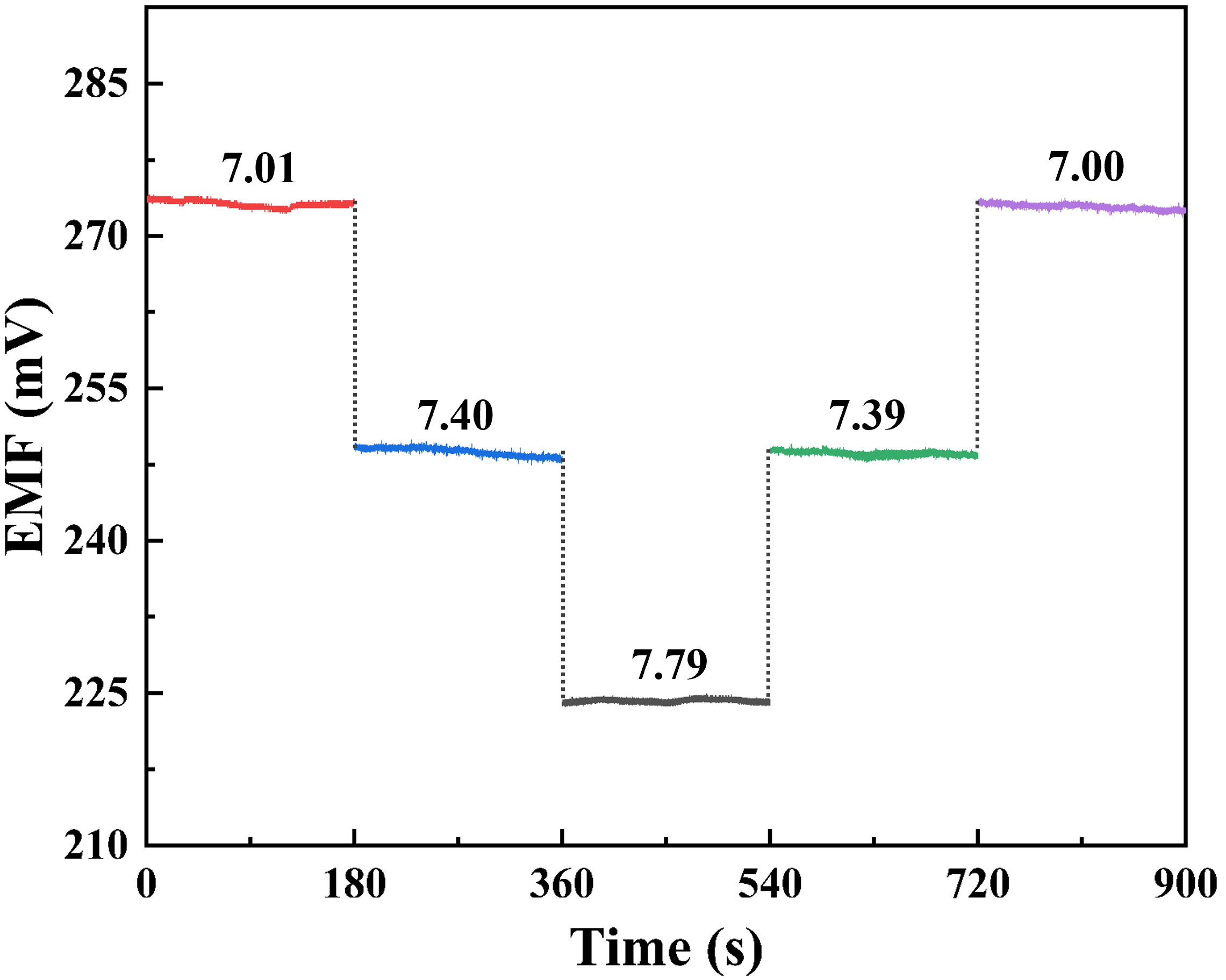


**Fig. S2: Dynamic potential response of the DLC-UME in solutions with different pH values.**
